# Trauma treatment outcomes for PTSD in refugee and asylum-seeking minors with uncertain residence status: a systematic review

**DOI:** 10.3389/fpsyt.2025.1715650

**Published:** 2026-01-14

**Authors:** Catarina Nahlén Bose, Miguel Diaz

**Affiliations:** Department of Health Sciences, The Swedish Red Cross University, Huddinge, Sweden

**Keywords:** asylum-seeker, child, cognitive behavioral therapy (CBT), eye movementdesensitization and reprocessing (EMDR), narrative exposure therapy (NET), post-traumatic stress disorder (PTSD), refugee, uncertain legal status

## Abstract

**Introduction:**

Refugee and asylum-seeking minors with an uncertain residence status are at risk of being refused treatment for PTSD due to claims that residence stability is required for a successful outcome.

**Objective:**

To synthesize research on the effectiveness of trauma treatment for PTSD in refugee and asylum-seeking minors with an uncertain residence status. Further objectives were to investigate whether there is any evidence that an uncertain residence status predicts treatment outcome and adherence to the treatment.

**Methods:**

Data searches were performed in Cinahl, Cochrane Library, PsychINFO and PubMed. A total of 741 articles were screened for eligibility, of which 23 were included in the systematic review.

**Results:**

Significant reductions in PTSD symptoms were reported in 17 of the 23 studies (2 RCTs and 16 NRSIs). The median effect size, reported in 11 studies was high, 0.97 (IQR 0.44 -1.23). No adverse effects were reported. The trauma treatment forms were mainly CBT, NET and EMDR. There was very little evidence to support whether an uncertain residence status would be a factor that predicts treatment outcomes or adherence to treatment. On average, adherence to treatment was 76%.

**Conclusion:**

Trauma treatment, such as CBT, NET and EMDR, for minors living under uncertain residence status can reduce levels of PTSD symptoms with a moderate to high effect size. The result thereby challenges the notion that residence stability is required for a successful trauma treatment outcome.

## 1 Introduction

About 48,8 million minors worldwide had been displaced due to conflict or violence at the end of 2024 of whom 19,1 million are refugees and asylum-seekers (1). Many of these minors have been exposed to traumatic events that can occur premigration, perimigration, and/or postmigration such as loss of family and separation, war, sexual violence, and torture. Stressors that minors can experience postmigration are stigma, discrimination, challenges navigating the healthcare system, and an uncertain residence status (2, 3). Minors often live under uncertain legal status in the countries they have migrated to, and the process for obtaining a permanent residence status is usually protracted leaving them living in uncertainty up to several years (4). The prevalence of mental disorders is higher in refugee and asylum-seeking minors compared to the general population. Previous reviews have reported prevalence of post-traumatic stress disorder (PTSD) from 19 to 53% (5, 6). Post-traumatic stress symptoms (PTSS) include symptoms such as re-experiencing the trauma, intrusion, e.g. having nightmares and flashbacks, avoidance of trauma-related stimuli, negative thoughts and feelings, increased arousal and reactivity e.g. hypervigilance, difficulty concentrating and sleeping, irritability and aggression (7). Uncertain residence status is one of the significant predictors for PTSS in refugee minors (8, 9). A previous study showed that minors with an uncertain residence status had a 76% higher risk of getting a PTSD diagnosis (9). Therefore, screening for and treating PTSD in this population is critical (10). Research has shown that PTSD in minors can result in abnormal development of the brain with a concomitant reduced emotion regulation and increased threat signaling activity as the child ages (11). In fact, a meta-analysis concluded that minors who had been exposed to childhood trauma displayed altered brain activity in functions such as cognitive and emotional processing regardless of a PTSD diagnosis when compared to controls (12). The long-term impact of PTSD can be fatal. A large cohort-study found that PTSD doubles the risk of suicide from the age of 14 (13). The detrimental effects of PTSD in minors constitute a strong foundation for appropriate screening and treatment. Furthermore, the Convention on the Rights of the Child states that children, regardless of their migration status, have a right to access healthcare and rehabilitation (Article 24), and that state parties should take actions to promote physical and psychological recovery for children that have been exposed to any form of violence or armed conflicts (Article 39) (14). Yet, there are children in asylum-processes that are refused treatment of PTSD by psychiatry units due to the belief that residence stability is a prerequisite for a successful treatment outcome (15). Therefore, the primary aim of this study was to synthesize research on the effectiveness of trauma treatment for PTSD in refugee and asylum-seeking minors (children and adolescents) with an uncertain residence status. Secondary objectives were to investigate, where studies explicitly measured and reported such associations, whether there is any evidence that an uncertain residence status predicts treatment outcomes and adherence. Accordingly, this systematic review addressed the following research questions: (1) In refugee and asylum-seeking minors living under uncertain residence conditions, what is the evidence that trauma-focused interventions reduce PTSD symptoms? (2) To what extent does residence status influence treatment outcomes and adherence, where this is examined in the included studies?

## 2 Methods

### 2.1 Design

This systematic review was conducted following the Preferred Reporting Items for Systematic Reviews and Meta-Analyses (PRISMA) guidelines (16).

### 2.2 Eligibility criteria

Inclusion criteria were studies evaluating trauma treatment targeting PTSD symptoms including minors < 18 years living under uncertain residence conditions as part of or the whole study sample. Original articles published in English or Swedish from 2004 to 2024 were considered for inclusion. The start year (2004) was selected based on initial scoping searches indicating that eligible studies first appeared from the mid-2000s, with the earliest included study published in 2004. By limiting the review to the last 20 years, we aimed to obtain relevant evidence to current clinical practice as well as the contemporary residence-status praxis of host countries. Exclusion criteria were studies that did not specifically address PTSD or lacked information about the participants’ residence status. Studies that only included adults were excluded.

### 2.3 Search strategy and data collection

**Figure 1:**
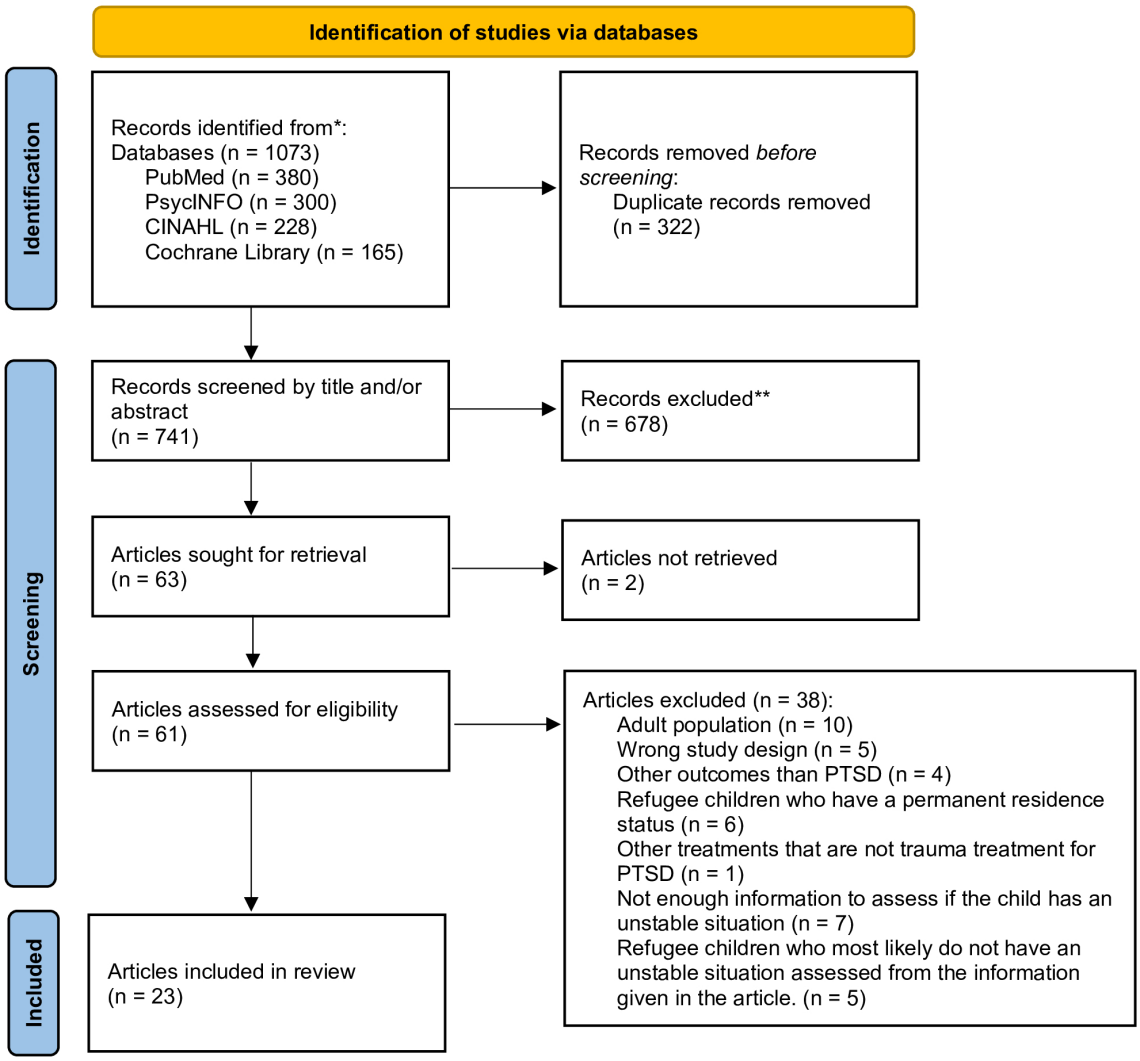
PRISMA flow chart of the screening process.

Comprehensive literature searches were conducted using the electronic databases PubMed, PsycINFO, Cinahl, and Cochrane Library. The search strategy combined subject headings and free text search words in four search blocks according to PICOC. Population: children/adolescents, Intervention: trauma intervention, Comparison: No predefined comparator was utilized in this study; Outcome: PTSD and Context: uncertain residence status (refugees/asylum seekers). A full account of the search strings is displayed in **Supplementary Material 1**. In total, 741 studies were screened for eligibility of which 23 studies were included in the review. The detailed screening process is displayed in **Figure 1**. All steps in the screening process were conducted by both authors, independently and blinded, via the tool Covidence. Any discrepancies were discussed to reach consensus.

### 2.4 Risk of bias assessment

Risk of bias was determined by using the revised tool to assess risk of bias in randomized trials (RoB2-tool) for the RCT-studies or risk of bias in non-randomized studies (Robins-1 V2) (17, 18). Data extraction from all included studies was performed independently by both authors, and any discrepancies were resolved by discussion.

### 2.5 Data extraction and data synthesis

Data extraction was performed by both authors and concerned amongst others, study characteristics, type of intervention, and outcome. Details of which data that were extracted are shown in **Tables 1**, **2**. The data was first tabulated and then synthesized regarding the outcome of the studies and adherence to the treatment. If any association between uncertain residence status and prediction on treatment were reported that was also synthesized. A meta-analysis was not performed due to the heterogeneity of the studies. The outcome of the studies was synthesized regarding statistical significance and effect size, if reported. The effect size, by using the standardized metric of Cohen’s d, was synthesized by summarizing effect estimates in median, interquartile range and range (19). As Hedge’s g adjusts for a smaller sample size and is very comparable to Cohen’s d, both estimates were summarized together. The intervention effect was also grouped according to type of intervention.

**Table 1:** Overview of population characteristics.

First author (year)	Country	N Partici-pants	Age (years)	Gender	Legal status	Baseline statistics of post-traumatic stress disorder (PTSD) or symptoms (PTSS) mean (SD)	Participants origin
Ehtnholt et al. (2005) (22)	UK	26	11-15	17 boys and 9 girls.	Asylum seekers	92.3% scored above the cut-off for probable PTSD.	Kosovo, Sierra Leone, Turkey, Afghanistan, Somalia
Fortuna et al. (2023) (23)	US	20	Mean: 15.8	15 boys and 5 girls	Asylum seekers	Child PTSD Symptom Scale: 21.67 (14.47) out of 51. No cut-off reported.	Guatemala, Honduras, El Salvador, Mexico
Garoff et al. (2019) (21)	Finland	18	9-17	16 boys and 2 girls	Asylum seekers and refugees	73% exceeded the cut-off for probable PTSD	Afghanistan & Iraq (mostly). Other origins not specified
Gotseva-Balgaranova et al. (2020) (24)	Bulgaria & Germany	15 (and their mothers)	6-11	7 boys and 8 girls	Asylum seekers and refugees	Trauma Symptoms Checklist for Young Children: 5.39 (1.13). No cut-off reported.	Iraq, Afghanistan, and Syria
Höhne et al. (2024) (26)	Germany	158	14-21	133 boys and 25 girls	Asylum seekers and refugees	The Child and Adolescent Trauma Screen self-report (CATS-self): 30.95 (2.13) out of 60. No cut-off reported	Afghanistan, Syria, Iran, and Eritrea (mostly). Other origins not specified
Hoskins et al. (2024) (25)	US	31 (and their caregivers)	10-18	7 boys and 24 girls	Undocumented caregivers and youth with immigrant backgrounds experiencing caregiver deportation	UCLA PTSD Index: 37.59 (16.70) Scores of 38 and above indicate clinically significant PTSD.	Latin America: Countries not specified
Lempertz et al. (2020) (27)	Germany	10	4-6	5 boys and 5 girls	Asylum seekers	60% scored above the cut-off for PTSD.	Syria, Afghanistan
Meyer DeMott et al. (2017) (28)	Norway	143	15-18	All boys	Asylum seekers	The Harvard Trauma Questionnaire (HTQ) post-traumatic symptom score: about 2.2. A score > 2 suggests probable clinical significance.	Afghanistan, Somalia, Iran, Palestine, Algeria, Western Sahara
Möhlen et al. (2005) (29)	Germany	10	10-16	6 boys and 4 girls	Refugees	60% fulfilled the diagnostic criteria for PTSD.	Kosovo
Onyut et al. (2005) (30)	Uganda	6	13-17	3 boys and 3 girls	Refugees	100% had moderate to severe PTSD according to the Composite Inter-national Diagnostic Interview: 14.3 (1.9).	Somalia
Oras et al. (2004) (31)	Sweden	13	8-16	3 boys and 10 girls	Asylum seekers	The Posttraumatic Stress Symptom Scale for Children: 61.8 (11.9) out of 128. No cut-off score reported.	Asia, Europe, and Africa. Countries not specified
Patel et al. (2024) (32)	US	122	4-19	76 boys and 62 girls	Unaccompanied migrant minors	34% reported experiencing clinically elevated symptoms of posttraumatic stress disorder, with 14.0% scoring within the “Probable PTSD”.	Mostly central America. Countries not specified
Pfeiffer et al. (2017) (33)	Germany	36	14-18	All boys	Unaccompanied young refugees	38.9% fulfilled the DSM-5 PTSD criteria. Inclusion criteria were mild to moderate severity of PTSS, as indicated by a total symptom score of > 15 on the CATS-self: 27.58 (7.88)	Afghanistan, Eritrea, Gambia, Pakistan, Albania, Syria, Somalia, Sudan, Iraq, Nigeria, Ghana
Pfeiffer et al. (2018) (34)	Germany	99	13-21	92 boys and 7 girls.	Unaccompanied young refugees	CATS-self: intervention group 29.97 (1.22), control group 31.85 (1.23). Inclusion criteria were mild to moderate severity of PTSS, as indicated by a total symptom score of > 19 on the CATS-self.	Mostly Afghanistan, Syria, Gambia, Somalia, Iran, Eritrea, Senegal, Iraq, Ethiopia, Pakistan, Angola, Nigeria, Ivory Coast, Ghana, Guinea, Guinea-Bissau, Kurdistan
Pfeiffer et al. (2019) (35)	Germany	50	14-19	47 boys and 3 girls	Unaccompanied young refugees	CATS-self: 29.91 (1.16). Inclusion criteria were mild to moderate severity of PTSS, as indicated by a total symptom score of > 19 on the CATS-self.	Top Eastern and Africa. Countries not specified
Rondung et al. (2022) (36)	Sweden	15	16-20	13 boys and 2 girls	Asylum seekers and refugees	The 13-item Children’s Impact of Event Scale (CRIES-13): 31.71 (12.06). Inclusion criteria were screening positive for PTSD.	Afghanistan and Eritrea
Ruf et al. (2010) (37)	Germany	26	7-16	14 boys and 12 girls	Asylum seekers and refugees	100% had been diagnosed with PTSD according to DSM-IV as it was an inclusion criteria. UCLA PTSD Index: Intervention group 43.3 (12.3), control group: 38.3 (8.6).	Turkey, Balkan, Syria, Chechnya, Russia, and Georgia
Said et al. (2020) (20)	UK	4	16-17	3 boys and 1 girl	Asylum seekers	100% were assessed to have severe PTSD and all scored above the clinical cut-off for PTSD.	Sudan, Vietnam, and Albania
Sarkadi et al. (2018) (38)	Sweden	46	14-18	43 boys and 3 girls	Asylum seekers	CRIES-8: 29.02 (6.33). Inclusion criteria were screening positive for PTSD as indicated by a score ≥ 17 on CRIES-8.	Afghanistan and Syria
Unterhitzenberger et al. (2015) (39)	Germany	6	16-18	4 boys and 2 girls	Asylum seekers and refugees	100% had moderate to severe symptom levels according to the Clinician Administered PTSD Scale for Children and Adolescents or the Posttraumatic Diagnostic scale.	Afghanistan, Somalia, and Iran
Unterhitzenberger et al. (2019) (40)	Germany	22	17-19	All boys	Asylum seekers	100% fulfilled diagnostic criteria of PTSD and the PTSD severity was high, CATS-self: 30.58 (7.16).	Mostly Afghanistan. Single participants from Eritrea, Gambia, Iran, Sierra Leone, Somalia, Sudan, and Syria
van Es et al. (2021) (41)	Netherlands	41	12-19	27 boys and 14 girls	Asylum seekers	82.8% had heightened symptoms of PTSD. CRIES-13: 42.59 (12.13). A score of ≥ 30 suggests an increased risk of PTSD.	Eritrea, Syria, and Afghanistan
van Es et al. (2023) (42)	Netherlands	10	15-18	8 boys and 2 girls	Asylum seekers	CRIES-13: 29.8 (13.3)	Eritrea and Syria

**Table 2:** Summary of Intervention Types and Treatment Outcomes.

First author (year)	Study type	Comparator	Intervention	Treatment duration and follow-up	Adherence to treatment	Residence status as a predictor of treatment outcome	Outcome measurement of PTSD and results (effect size reported when reported in the study)
Ehntholt et al. (2005) (22)	Non-randomized controlled study	Waiting-list control group with PTSD	Group-based CBT.	6 weekly sessions. 60 min each. 2-month follow-up	Not explicitly reported	Not reported	The 13-item Children’s Impact of Event Scale (CRIES-13) Significant difference between groups in overall PTSD symptom severity (p = 0.003). Significant reduction in overall PTSD symptoms within intervention group (p = 0.011) and a non-significant increase within control group (p = 0.073).
Fortuna et al. (2023) (23)	Non-randomized mixed methods approach, within-subject	U.S.-born children with PTSD	Mindfulness-based CBT integrating religiosity/spirituality	12 weekly sessions. No follow-up	100%	Not reported	The Child PTSD Symptom Scale (CPSS) and The Posttraumatic Cognitions Inventory The unaccompanied immigrant children showed significant improvement in PTSD symptoms (p<0.05, Cohen’s d=1.15) and posttraumatic cognitions (p<0.05, Cohen’s d=0.6), particularly improvements in negative cognitions about the world (p<0.05, Cohen’s d=0.82). The U.S.-born subsample showed significant improvement in PTSD symptoms (p < 0.05, Cohen’s d=0.34), combined PTCI cognitions (p < 0.01, Cohen’s d=0.47) and Negative Cognitions about the Self (p <.01, Cohen’s d=0.47).
Garoff et al. (2019) (21)	Within-subject mixed methods approach (pre-post measurements)	None	Group-based intervention as part of a SCM (first-level intervention, psychoeducation, coping strategies, and social support).	10 weekly sessions. 90 min each. No follow-up	90%	Not reported	CRIES-13 No significant statistical changes detected in PTSD symptoms, p = 0.23, 95% CI diff [–2.88, 10.68] Hedge’s g = 0.35.
Gotseva-Balgaranova et al. (2020) (24)	Within- subject (pre-post measurements)	None	Parent-child EBTS	9 weekly sessions. No follow-up	94%	Not reported	Trauma Symptoms Checklist for Young Children (TSCYC) – parent assessment Children Stress Checklist (cPC) -based on two subscales from TSCYC - self assessment No significant reduction in PTSD total scores (TSCYC: p=0.17, Cohen’s d=0.44). However, there were significant reductions in specific PTSD symptoms: Intrusion (cPC: p=0.05, Cohen’s d=0.44), arousal (TSCYC: p=0.09, Cohen’s d= 0.47), depression (TSCYC: p=0.09, Cohen, 0.48), and dissociation (TSCYC: p=0.097, Cohen’s d= 0.46).
Höhne et al. (2024) (26)	Cluster-randomized control trial	Treatment as usual (regular access to routine healthcare services)	Culturally sensitive SCM, including: Watchful waiting (low symptoms) Smartphone app (moderate symptoms) Group intervention (moderate-severe symptoms) Individual psychotherapy (high symptom severity)	12 weekly sessions. 3 and 6-month follow-up	52%	Not reported	The Child and Adolescent Trauma Screen self-report (CATS-self) No significant difference between intervention and control groups at any of the follow-ups. Both groups had significant within-group reductions in PTSD symptoms up to 6-months (p<0.05, Cohen’s d=0.243).
Hoskins et al. (2024) (25)	Within-subject (pre-post measurements)	None	Multifamily group therapy integrating trauma-informed interventions, resilience strategies, and positive psychology tailored for Latinx families.	10 weekly sessions. 90 min each. No follow-up	>90%	Youth who experienced caregiver deportation showed larger reductions in PTSD symptoms compared to those who had not experienced caregiver deportation.	The Trauma Symptom Checklist for Children (TSCC) and the UCLA PTSD Index. Significant reductions in PTSD symptoms (TSCC and UCLA PTSD Index: p<0.001).An examination of the means indicated a larger reduction in the severity of PTSD symptoms (UCLA PTSD Index) for Latinx youth who experienced a caregiver’s deportation (Mdiff = 24) compared to Latinx youth with no history of caregiver deportation (Mdiff = 9.1)
Lempertz et al. (2020) (27)	Within-subject (pre-post measurements)	None	EMDR-based group therapy using a storytelling approach	5 daily sessions. 60 min each. 3-month follow-up	70%	Not reported	15 questions from the 100-item Child Behavior Checklist for ages 1½–5 years Significant reduction in PTSD symptoms at post-treatment (p=0.018, Cohen’s d = 0.93 and follow-up (p=0.034, Cohen’s d = 0.81) when assessed by the teachers. However, when assessed by parents there were no significant decrease in PTSD symptoms over the follow-up period (p=0.057).
Meyer DeMott et al. (2017) (28)	Non-randomized controlled trial	Life as Usual: standard activities including education, soccer, dance activities at the reception center, without expressive arts.	Group-based expressive arts therapy focusing on stabilization, anxiety, and stress management, emotional regulation, and trauma education.	10 sessions. 2 sessions weekly. 90 min each. 5, 10, and 25-month follow-up	92%	Not reported Most had received an asylum decision after 1–2 years, where 34% in the intervention group and 25% in the control group was granted asylum. Uncertainty could therefore not explain any differences between the groups.	The Harvard Trauma Questionnaire (HTQ) post-traumatic symptom score There was a significant time by group interaction (p =0.042) in favour of the intervention group for reduced PTSD symptoms, but no significant group differences at any time point (p= 0.053).
Möhlen et al. (2005) (29)	Within-subject (pre-post measurements)	None	Multimodal psychosocial intervention. Individual, family, and group sessions including psychoeducation, trauma and grief-focused therapy, creative and relaxation techniques, storytelling, and guided imagery.	12 weekly sessions. 2–3 hours each. No follow-up	100%	Not reported	HTQ post-traumatic symptom score Significant reduction in PTSD symptoms (p=0.018).
Onyut et al. (2005) (30)	Within-subject (pre-post measurements)	None	KIDNET	4–6 sessions. 1–2 hours each. 9-month follow-up	100%	Not reported	The Composite International Diagnostic Interview (CIDI) version 2.1 Sections K Significant reduction in PTSD symptoms from pre-post-treatment as well as 9-month follow-up (p<0.01).
Oras et al. (2004) (31)	Within-subject (pre-post measurements)	None	EMDR combined with conversational therapy (for adolescents) or play therapy (for children younger than 13)	5–25 sessions- 1–2 sessions per week. No follow-up	Not explicitly reported	Best effect of the treatment was reported in children with stable family situations.	The Posttraumatic Stress Symptom Scale for Children (PTSS-C) Significant reduction PTSD symptoms (p<0.01). The significant reduction was found in all but one participant.
Patel et al. (2024) (32)	Within-subject (pre-post measurements)	None	TF-CBT including culturally adapted components	~ 9 sessions. No fixed duration stated. No follow-up	42%	Not reported	CATS-self and CATS-caregiver report (CATS-care) Significant reduction in overall trauma symptoms (CATS-self: p<.001, Cohen’s d = -1.03 and CATS-care: p = .008, Cohen’s d = -0.79).
Pfeiffer et al. (2017) (33)	Within-subject (pre-post measurements)	None	TF-CBT	6 weekly sessions, 90 min each. No follow-up	81%	Not reported	CATS-self Significant reductions in overall PTSS symptom scores (p=0.001, Cohen’s d =0.97). Specific posttraumatic symptom reduction occurred in re-experiencing (p=0.001, Cohen’s d =0.84), avoidance (p=0.041, Cohen’s d =0.45), negative cognitions and mood symptoms (p=0.001, Cohen’s d =0.87) but not in hyperarousal (p=0.104).
Pfeiffer et al. (2018) (34)	Randomized controlled trial	Usual care (standard child welfare support without structured trauma intervention)	TF-CBT	6 weekly sessions. 90 min each. 2-month follow-up	74%	Not reported	CATS-self and CATS-care Significant reduction in PTSD symptoms (CATS-self) post-treatment in favour of the intervention group as indicated by a significant interaction between group and time (p = 0.003). The between-group effect size post-treatment controlled for baseline values was Cohen’s d =0.33. Within-group analysis revealed an effect size of Cohen’s d=0.61 for the intervention group and an effect size of Cohen’s d=0.15 for the control group. No significant interaction effect of group and time were found for caregiver-rated PTSS.
Pfeiffer et al. (2019) (35)	Within-subject (pre-post measurements) Sub-study from a randomized controlled trial analysing only the intervention group.	Usual care (control group not included in analysis)	TF-CBT	6 weekly sessions. 90 min each. 3-month follow-up	74%	Country of origin (particularly Afghanistan) predicted poorer treatment response. The article discusses that the finding might be caused by the threat of being deported as overshadowing the benefit from the intervention.	CATS-self and CATS-Care Self-reported PTSS decreased significantly (pre to post change (p<0.001, Cohen’s d = 0.62); pre to 3-months follow-up change (p<0.001, 0.64); post to 3-month follow-up change (p=0.47, Cohen’s d = 0.11). When PTSS was measured by caregiver there were no statistically significant reduction in PTSS.
Rondung et al. (2022) (36)	Pilot randomized control trial	Waiting list	TF-CBT	7 weekly sessions, 2 hours each. 4-month follow-up	43%	Not reported	CRIES-13 No statistical significance is reported. Pilot data indicated decreased PTSD symptoms at post-intervention and further reductions at follow-up. Only numerical data is presented. Baseline 31.71 (SD12.06), 4-months follow-up 19.43 (SD 9.78).
Ruf et al. (2010) (37)	Randomized control trial	Waiting list	KIDNET	8 sessions. 90–120 min each. 1, 6 and 12-month follow-up	92%	Not reported	The UCLA PTSD Index Significant reduction in PTSD symptoms sustained at 6-month follow-up compared to controls (p<0.01; Hedge’s g 1.9). Significant reduction in PTSD symptoms within intervention group (Hedge’s g 1.8) at 12-month follow-up.
Said et al. (2020) (20)	Within-subject (pre-post measurements)	None	NET	9–20 sessions, once a week. No follow-up	75%	Not reported	CRIES-8 (8 item version) The reliable change index indicated reliable improvements for 3 out of 4 participants at the 5% significance level. Two participants fell below clinical PTSD cut-off post-intervention compared to scoring above clinical cut-off for PTSD before treatment.
Sarkadi et al. (2018) (38)	Within-subject (pre-post measurements)	None	Group-based TF-CBT and TRT	5 weekly sessions, 90–120 min. No follow-up	43.5%	Not reported	CRIES-8 Significant reduction in PTSD symptoms post-intervention (p=0.017).
Unterhitzenberger et al. (2015) (39)	Within-subject (pre-post measurements)	None	TF-CBT	12–28 sessions, 100 min. No follow-up	Not explicitly stated	Not reported An important focus of the intervention was seen in enhancing a feeling of safety.	The Clinician Administered PTSD Scale for Children and Adolescents (CAPS-CA) or the Posttraumatic Diagnostic Scale (PDS) Significant reduction in PTSD symptoms (CAPS-CA and PDS: p<0,001). Clinically meaningful improvement in all the participants CAPS-CA and PDS.
Unterhitzenberger et al. (2019) (40)	Within-subject (pre-post measurements)	None	TF-CBT	15 sessions, 100 min. 6-week and 6-month follow-up	84%	At 6-month follow-up, participants whose asylum claims had been rejected showed increased PTSD symptoms, but it was not statistically significant from the group that had not yet received a decision or had a granted asylum request.	The Diagnostic Interview for Mental Disorders in Childhood and Adolescence (Kinder-DIPS) CATS-self and CATS-care Significant reduction in PTSD symptoms (CATS-self) post-intervention, as well as 6-week, and 6-month follow-up (Cohen’s d=1.23). Significant reduction in PTSD symptoms measured by proxy (CATS-care) at 6-month follow-up (Cohen’s d=2.38). Caseness of PTSD (Kinder-DIPS) fell significantly from 100% at 6-week to 16% at 6-month follow-up, a recovery rate of 84%
van Es et al. (2021) (41)	Within-subject (pre-post measurements)	None	TF-CBT, KIDNET and EMDR	8 sessions, 90min. No follow-up	Not explicitly stated	Not explicitly analyzed. However, uncertain residence status was identified as a stressor affecting adherence. The therapists sometimes deviated from the protocol to address the uncertainty.	CRIES-13 Significant reduction in PTSD symptoms (p<0.001, Cohen’s d=1.32).
van Es et al. (2023) (42)	Within-subject mixed methods approach (pre-post measurements)	None	TF-CBT, KIDNET and EMDR	8 sessions, 80 min. 1-month follow-up	Not explicitly stated	The feasibility of the study was influenced by factors related to the setting and population, including news concerning asylum status.	CRIES-13 The reliable change index did not show statistically significant clinical changes at post-treatment or follow-up.

CBT: Cognitive Behavioral Therapy. EBTS: Evidence-Based Trauma Stabilisation. SCM: Stepped Care Model. KIDNET: Narrative Exposure Therapy for children. NET: Narrative Exposure Therapy. TRT: Teaching Recovery Techniques. EMDR: Eye Movement Desensitization and Reprocessing. TF-CBT: Trauma-Focused Cognitive Behavioral Therapy.

## 3 Results

### 3.1 Risk of bias

**Figure 2:**
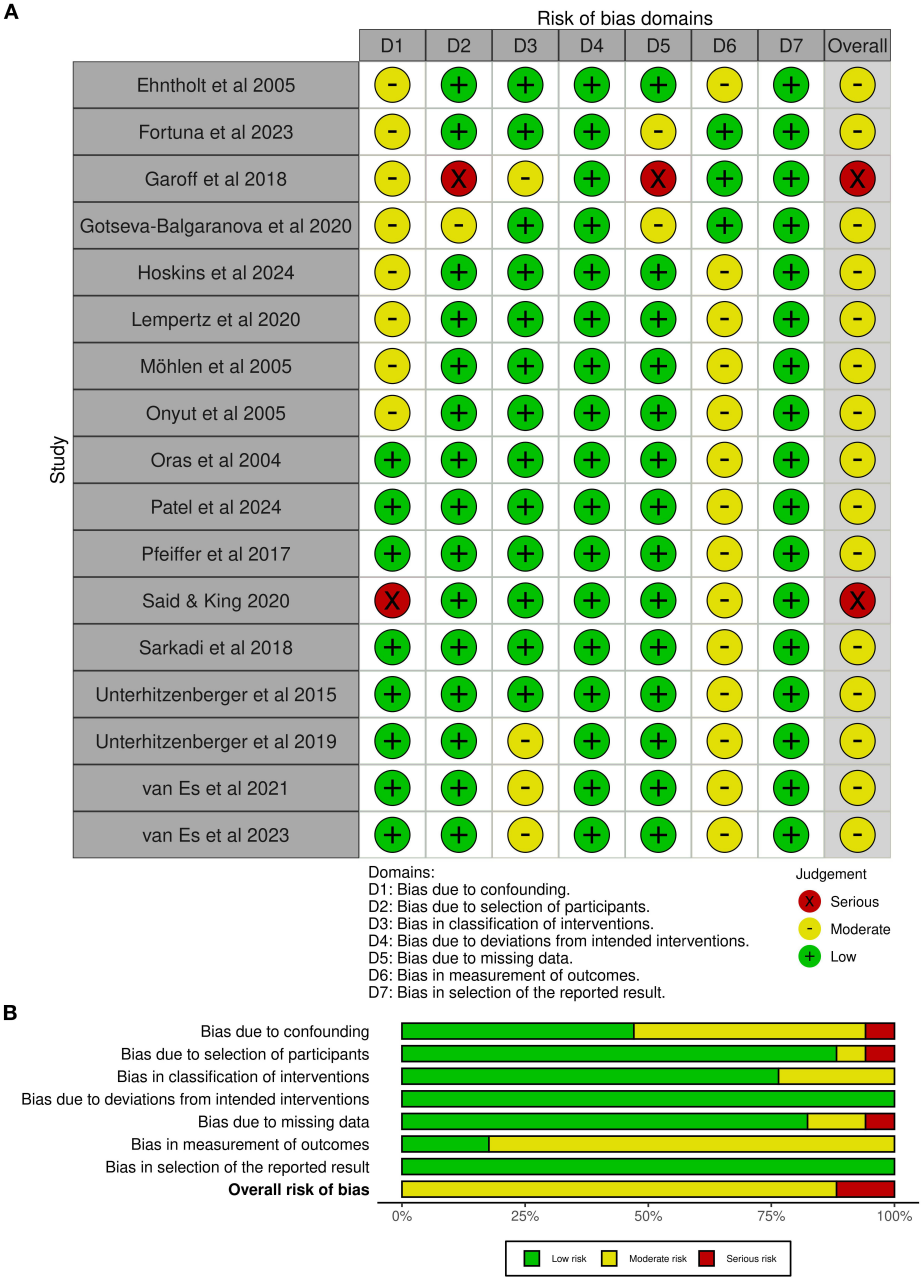
**(A)** ROBINS-I traffic light plot. **(B)** ROBINS-I summary plot.

**Figure 3:**
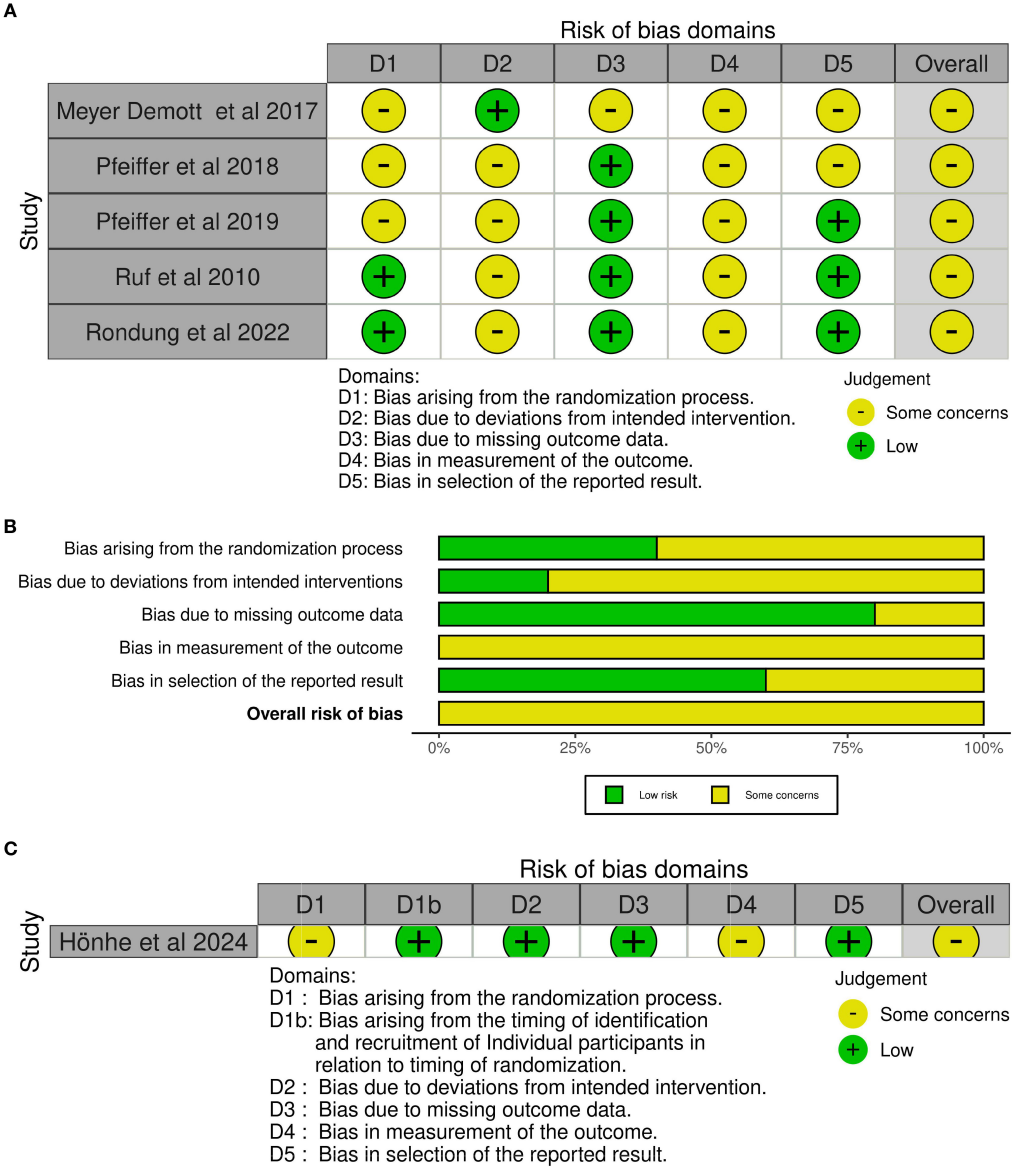
**(A)** ROB2 traffic light plot. **(B)** ROB2 summary plot. **(C)** ROB2-Cluster traffic light plot.

Seventeen studies implemented a non-randomized quasi-experimental design with within-subject measurements pre- and post-treatment. Two of them were assessed as having a serious risk of bias in respect to confounding (20), as well as selection of participants, and missing data (21). The remaining non-randomized studies were assessed as having a moderate risk of bias (**Figures 2A, B**). Six studies implemented a randomized controlled design, where one was a cluster randomized trial. All of them were assessed as having some concerns about risk of bias, particularly regarding the randomization process, deviation from intended intervention, as well as measurement of outcome (**Figures 3A–C**).

### 3.2 Study characteristics

This systematic review included 23 studies published between 2004 and 2024. The publication timeline was uneven, with only a small number of studies in the mid-2000s and a larger concentration of studies published during the last decade (20–42). (**Table 1**) Studies were predominantly conducted in Europe, particularly Germany (n=10), Sweden (n=3), the United Kingdom (n=2), and the Netherlands (n=2), with single studies from Finland, Norway, and Bulgaria (in collaboration with Germany). Three studies were conducted in the USA and one in Uganda.

### 3.3 Participants, outcome measurement tools and treatment settings

The total number of participants varied considerably across studies, ranging from as few as 4 (20) to 143 participants (28). Most studies involved adolescents, typically ranging from 12 to 18 years of age, although the overall age range was 4 to 21 years. Few studies included children under 7 years (24, 27, 32). Participants that exceeded the clinical cut-off for PTSD ranged from 34 to 100% at baseline when reported in the studies. The tools for measuring symptoms of PTSD varied between the studies. In total, there were 15 different tools where some studies applied two tools, for example one administered to caregivers and one administered to the minors for self-assessment. The tools used most frequently, n=5 respectively, were the 13-item Children’s Impact of Event Scale (CRIES-13) and the Child and Adolescent Trauma Screen self-report (CATS-self) (**Table 2**). Gender distribution showed a clear predominance of male participants across most studies, with several studies involving exclusively male samples (e.g (28, 33, 40)). Some studies, however, reported a more balanced gender distribution, or predominantly female samples (25, 31, 32). Participants originated from diverse regions, primarily Afghanistan, Syria, Iraq, and African countries such as Eritrea and Somalia. Studies conducted in the USA mostly involved refugees from Central and Latin American countries.

The studies were conducted in diverse treatment settings, explicitly tailored to the population’s unique circumstances and needs. Most interventions occurred in specialized mental health or psychotherapeutic outpatient clinics (e.g (26, 40)), child and adolescent welfare facilities (33–35), or community-based settings like schools, residential care homes, and asylum centers (22, 23, 38). Some studies employed outreach approaches, delivering therapy in the participants’ familiar environments, such as accommodations units or location chosen by the participants themselves (21, 41, 42). The involvement of intercultural mediators, interpreters, or multilingual therapists was explicitly reported in a number of studies (24, 41, 42). A few studies explicitly described treatment within schools or daycare centers (22, 23, 27).

### 3.4 Intervention types, intervention duration and follow-up

There was a variation of trauma treatment types in the studies, and they were grouped accordingly: 1) Cognitive Behavioural Therapy (CBT) (n=10), 2) Narrative Exposure Therapy (NET) or KIDNET (n=3), 3) Eye Movement Desensitization and Reprocessing (EMDR) or EMDR-combined (n=4), 4) Interventions with various forms of trauma treatment elements including psychoeducation, expressive arts and multi-family therapy (n=5). Intervention durations varied widely, typically involving weekly sessions lasting between 60 to 120 minutes. The most common formats included 6 to 10 weekly sessions. Shorter interventions consisted of intensive daily sessions lasting approximately one week (27), whereas more extended interventions involved weekly treatments lasting several months (29, 40). Follow-up measurements were conducted in approximately half of the studies (11 out of 23) whereas the rest only had post-intervention measurements. Follow-up ranged from short periods of 2 weeks to intermediate follow-up of 2–3 months or long-term follow-up to 25 months post-treatment (28).

### 3.5 Treatment outcomes

**Figure 4:**
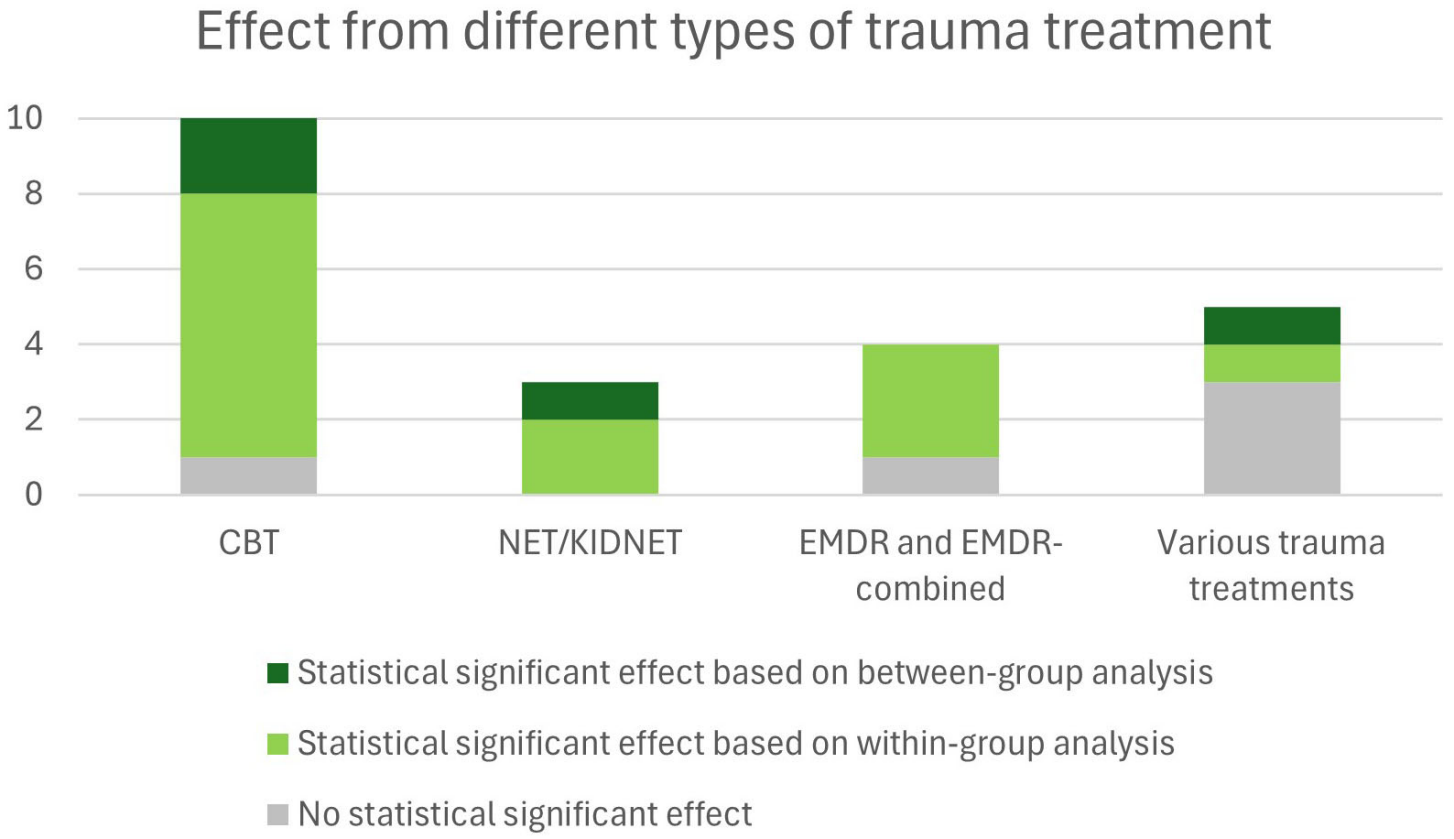
Effect from different types of trauma treatment.

**Figure 5:**
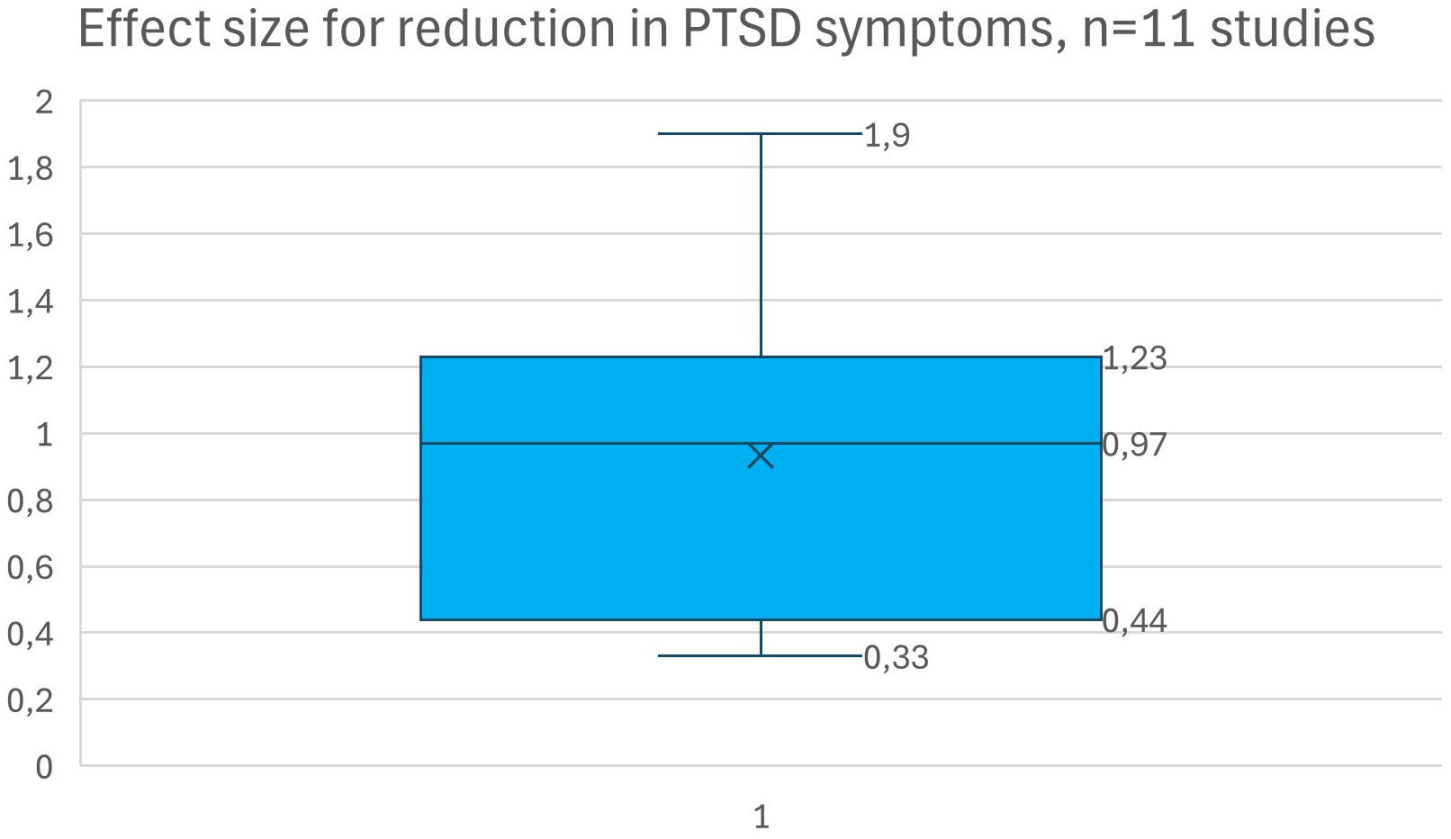
Effect size in Cohen’s d for reduction in PTSD symptoms, n=11 studies.

A detailed description of trauma treatment outcomes is presented in **Table 2**, and a summary for the different treatment types is displayed in **Figure 4**. Significant reductions in PTSD symptoms were reported in 17 of the 23 studies (2 RCTs and 16 NRSIs). Of the studies that did not show any statistically significant reductions in the total level of PTSD symptoms there was one study that reported significant reductions in specific PTSD symptoms, such as intrusion and arousal (24), and one RCT-study that reported significant reductions when a within-group analysis was performed (26). Six studies reported maintained effects at follow-ups from 3 to 25 months (26–28, 30, 34, 35) whereas one study that showed significant short-term improvements did not maintain the effect at follow-up (22). Some studies only reported qualitative improvements without statistically significant quantitative results (21, 25, 42). Eleven studies reported effect sizes, in Cohen’s d (n=9) or Hedge’s g (n=2), where the median effect size was high, 0.97 (IQR 0.44 -1.23). The total range of effect size was between moderate to high (0.33 to 1.9) (**Figure 5**).

#### 3.5.1 Cognitive behavioral therapy

Ten studies reported using CBT (n=8 TF-CBT, n=1 school-based CBT, n=1 mindfulness-based CBT). Of these studies, nine reported statistically significant reductions in PTSD symptoms (22, 23, 32–35, 38–40). Three studies showed a sustained effect from two to six months after treatment (34, 35, 40). One study did not perform a statistical analysis and only reported a total score decrease in PTSD symptoms up to four months after treatment (36).

#### 3.5.2 Narrative exposure therapy

Three studies used NET where two used the child-friendly version KIDNET and one NET. All studies reported significant reductions in PTSD symptoms post-intervention (20, 30, 37) that was maintained after 9 to 12 months in two of the studies (30, 37).

#### 3.5.3 Eye movement desensitization and reprocessing

One study used EMDR as a single intervention (27). Two studies used EMDR in combination with TF-CBT and KIDNET (27, 41, 42). However, EMDR composed the main treatment (47% of the sessions), KIDNET (40% of the sessions) and TF-CBT (12% of the sessions) (41). One study used EMDR in combination with conversational therapy for adolescents or play therapy for children younger than 13 (31). Three out of four studies reported statistically significant reductions post-intervention in PTSD symptoms (27, 31, 41) where one study showed a sustained effect after 3 months (27).

#### 3.5.4 Interventions with various forms of trauma treatment elements

Five studies had various forms of trauma treatment elements such as Stepped Care Model (SCM) (21, 26), parent-child evidence-based trauma stabilisation (EBTS) (24), multifamily group therapy (25) and expressive arts intervention (28). The studies with multifamily group therapy and expressive arts intervention showed significant reductions in PTSD symptoms (25, 28) where the latter had a significant time by group interaction over a 25-months period.

### 3.6 Residence uncertainty and prediction of treatment outcome and adherence

Residence uncertainty was addressed with considerable variability where some studies provided detailed descriptions of the asylum context and some studies mentioned uncertainty more generally. All studies included either asylum seekers or refugees. Most studies did not measure residence status as a factor that could influence treatment outcome. One study (40) reported subgroup comparisons relevant to residence stability with no significant differences at follow-up, and two others (25, 31) mentioned residence stability-related differences in treatment only descriptively and without a statistically tested moderated effect of treatment. However, some of the studies discussed uncertainty related to awaiting asylum decisions and the fear of rejection as major contextual stressors potentially affecting participants’ psychological well-being and treatment outcomes (25, 28, 31, 35, 41, 42). One study reported the best treatment outcomes in a subgroup of children living in stable family life situations. However, almost all children in the study, regardless of life situation reported improvements in treatment outcomes (31). Another study divided the sample in two subgroups with either minors with rejected asylum request or minors waiting for asylum hearing, waiting for asylum decision, or who had asylum granted at 6-months follow-up and found that the latter subgroup had maintained improvements even though some of them had not yet received a decision, whereas the subgroup that had its asylum request rejected showed more symptoms of PTSD. However, the difference was not statistically significant (40). Regarding adherence to treatment, few studies explicitly connected residence uncertainty with higher dropout rates or lower adherence, explicitly describing participants who left treatment prematurely due to uncertainty-related stressors, legal complications, or relocation (25, 28, 31, 40–42). Overall, reported in 18 studies, the adherence to the intervention was on average 76% ranging from 43,5 to 100%.

## 4 Discussion

This review specifically focused on refugee and asylum-seeking minors with an uncertain residence status and the effectiveness of PTSD treatment. In general, the results show that trauma treatment reduces PTSD symptoms despite an uncertain residence status, with about a fourth of the studies showing maintained effects at follow-ups from 3 to 25 months. Furthermore, no adverse effects were found. Thereby, the results somewhat challenge the notion that residence stability is necessary for a successful treatment outcome (15). Other reviews on refugee- and asylum-seeking minors, although not solely focusing on minors with an uncertain residence status, have also shown that trauma treatment can be effective in reducing PTSD symptoms in this population (43, 44). Although the number of publications increased in the later part of the 2004–2024 window, we did not observe a clear temporal trend in treatment effects. Reductions in PTSD symptoms were reported across the period with a higher number of reports published in the last decade. In our study, the specific type of treatments that successfully reduced PTSD symptoms in minors with an uncertain residence status were predominately conventional trauma treatments such as CBT, NET or KIDNET, and EMDR or EMDR combined with TF-CBT, KIDNET, conversational therapy, or play therapy. The results are in line with what has been found to be effective in the general paediatric population (45, 46). Further, our results show that other forms of trauma treatments, like multifamily group therapy and expressive arts intervention are also effective in reducing PTSD symptoms in minors with an uncertain legal status. Other research indicates the evidence for art therapy in reducing symptoms of PTSD to be inconclusive. A previous systematic review found the evidence to be insufficient in refugee children and youth (47), whereas another systematic review and meta-analysis did find creative arts-based interventions to significantly reduce symptoms of PTSD in children and adolescents that had been exposed to traumatic events (48). Multifamily group therapy seems to be a novel treatment for minors with uncertain residence status. A conceptual analysis has discussed potential benefits of such a treatment in the context of intergenerational psychotrauma (49), which may also be a phenomenon refugee and asylum-seeking minors are exposed to (50).

The studies in this review mainly included children over seven years of age and the majority were teenagers. Hence, the evidence for younger refugee and asylum-seeking children with an uncertain residence status is scarce. The lack of younger children in trauma treatment studies has been found in other systematic reviews as well, for instance in trauma treatment for children who survived torture (51). This underscores a knowledge gap that needs to be addressed in future research.

Based on our findings, we cannot unequivocally argue that residence status would negatively impact trauma treatment outcomes or adherence to treatment. In line with this, a study exploring factors influencing utilization of TF-CBT amongst unaccompanied young refugees showed that residence status did not impact intention to utilize or the actual utilization of mental health care (52). However, there could be cultural and structural barriers to mental health care, such as fear of discrimination and stigmatization as well as lack of information about previous health issues prior to the trauma. Hence, it is not always the refusal of the health care provider or a deportation decision that leaves the minors untreated for PTSD (53). That being said, it is the health care provider who is responsible for providing care and treatment for refugee and asylum-seeking minors as regulated in the Convention on the Rights of the Child for the states who have ratified the act (14). The results of this review support that trauma treatment is effective for minors with an uncertain residence status, and it could therefore be argued that such treatment should be offered to them to alleviate suffering regardless of whether a state has ratified the Convention on the Rights of the Child.

### 4.1 Summary of study limitations and strengths

Several limitations must be considered when interpreting the feasibility and efficacy of PTSD treatment in minors from the studies in this review. First, residence status was rarely tested as a predictor of treatment response, which hinders drawing conclusions about its moderating impact. Second, most studies reported a lack of control groups, particularly those which implemented a design with pre-post measurements (**Table 2**). Third, a small sample size was commonly reported, with some studies including as few as four participants (**Table 1**). Fourth, approximately half of the studies did not follow up treatment outcome and most of those that did include a follow-up period, re-evaluated participants within six months of initial treatment. Only two studies included follow-up evaluations that extended over a year (**Table 1**). Fifth, several studies explicitly discussed potential biases arising from self-reported measures, lack of blinding, and potential cultural and language barriers. Sixth, although developmental stage is likely to matter, most studies in this review consisted of broad age ranges. Populations varied between 4–21 years, with most samples including minors between 12–18 years. Some studies adapted the interventions to fit the age of their samples (27, 31), but none of the included studies reported PTSD outcomes stratified by narrower developmental periods such as, preschool, school-age or adolescence, limiting developmentally specific conclusions. Seventh, there was heterogeneity in the studies. For instance, the measurement tools for symptoms of PTSD varied. However, there is no gold standard for measuring symptoms of PTSD. Several of the tools were based on the DMS-5 criteria for PTSD and demonstrate good reliability, however, measuring symptoms of PTSD in minors is more challenging (54, 55). Lastly, generalizability of the results is limited due to gender imbalance and origin of participants. Most samples included predominantly (some exclusively) male participants, and participants from Afghanistan, Syria and Eritrea (**Table 1**). On the other hand, the studies reviewed show certain methodological strengths. First, most studies reported a moderate to high adherence to treatment. Second, most of them used standardized instruments to measure PTSD. Third, the majority implemented manualized, replicable treatments such as CBT, KIDNET and EMDR (**Table 2**).

### 4.2 Conclusions

Trauma treatment, such as CBT, NET, and EMDR in minors living with uncertain residence status can reduce levels of PTSD symptoms with a moderate to high effect size and with no adverse effects. Moreover, the results could not conclude that residence status, as a sole factor, would predict the treatment outcome or adherence to treatment. These results thereby challenge the notion that residence stability is required for a successful trauma treatment outcome. Future research could focus on including younger children, an equal distribution of participants from both sexes, stratifying results based on developmental age, as well as longer follow-ups. The findings discussed in this review give health care providers a palette of different evidence-based trauma treatments that can be implemented to minors under uncertain residence status depending on what trauma treatment is practiced in their clinic and what is suitable for the specific context the minor is in.

## Data Availability

The original contributions presented in the study are included in the article/**Supplementary Material**. Further inquiries can be directed to the corresponding author.
