# Supplementary material for: Trauma treatment outcomes for PTSD in refugee and asylum-seeking minors with uncertain residence status: a systematic review
Source: Front Psychiatry. 2026 Jan 14;16:1715650. doi: 10.3389/fpsyt.2025.1715650 (PMC12847239; doi:10.3389/fpsyt.2025.1715650)
Supplement: Supplementary file 1 [file Supplementaryfile1.docx]

Supplementary Material 1

**Search strings**

| **Search blocks** | **Search terms in each database** |
| --- | --- |
| Children | Cinahl (free search terms were search in Title and Abstract in all blocks)  Child* OR (MH "Child") OR minor* OR (MH "Minors (Legal)") OR adolescent OR (MH "Adolescence")  Cochrane Library  Child* OR adolescent* OR minor* Youth* OR Teen* OR Teenager.  PubMed  Child* OR adolescent* OR minor* Youth* OR Teen* OR Teenager.  PsychInfo  tiab(child*) OR tiab(minor*) OR MAINSUBJECT.EXACT("Early Adolescence") OR tiab(adolescent*) |
| Trauma intervention | Cinahl  “Trauma treatment” OR intervention* OR (MH "Psychosocial Intervention") OR (MH "Intervention Trials") OR (MH "Early Intervention") OR (MH "Crisis Intervention") OR treatment* OR (MH "Treatment Delay") OR “rehabilitation” OR (MH "Rehabilitation, Pediatric") OR (MH "Rehabilitation") OR (MH "Rehabilitation, Psychosocial") OR “Cognitive Behavioral Therapy” OR (MH "Cognitive Therapy") OR CBT OR “trauma focused cognitive behavioral therap*" OR “TF-CBT” OR "Eye Movement Desensitization and Reprocessing" OR (MH "Eye Movement Desensitization and Reprogramming") OR EMDR OR “Narrative exposure therapy” OR NET OR KIDNET  OR "prolonged exposure" OR "Psychosocial Intervention" OR “Psychodynamic intervention” OR (MH "Psychotherapy, Psychodynamic") OR "Mind-Body Therap*" OR (MH "Mind Body Techniques") OR "trauma releasing exercise*"  Cochrane Library  “Trauma treatment” OR intervention* OR treatment* OR rehabilitation OR “Cognitive Behavioral Therapy” OR CBT OR “trauma focused cognitive behavioral therap*" OR TF-CBT OR "Eye Movement Desensitization and Reprocessing" OR EMDR OR “Narrative exposure therapy” OR NET OR KIDNET OR "prolonged exposure" OR "Psychosocial Intervention" OR “Psychodynamic intervention” OR "Mind-Body Therap*" OR "trauma releasing exercise*"  PubMed  “Trauma treatment” OR intervention* OR treatment* OR rehabilitation OR “Cognitive Behavioral Therapy” OR CBT OR “trauma focused cognitive behavioral therap*" OR TF-CBT OR "Eye Movement Desensitization and Reprocessing" OR EMDR OR “Narrative exposure therapy” OR NET OR KIDNET OR "prolonged exposure" OR "Psychosocial Intervention" OR “Psychodynamic intervention” OR "Mind-Body Therap*" OR "trauma releasing exercise*"  PsychInfo  (tiab("trauma treatment") OR MAINSUBJECT.EXACT("Trauma Treatment") OR tiab(intervention*) OR MAINSUBJECT.EXACT("Intervention") OR tiab(treatment*) OR MAINSUBJECT.EXACT("Treatment") OR tiab(rehabilitation) OR MAINSUBJECT.EXACT("Psychosocial Rehabilitation") OR tiab("Cognitive Behavioral Therapy") OR MAINSUBJECT.EXACT("Cognitive Behavior Therapy") AND pd(20040101-20241231)) OR (tiab(CBT) OR tiab("trauma focused cognitive behavioral therap*") OR MAINSUBJECT.EXACT("Trauma-Focused Cognitive Behavior Therapy") OR tiab(TF-CBT) OR tiab("Eye Movement Desensitization and Reprocessing") OR MAINSUBJECT.EXACT("Eye Movement Desensitization Therapy") OR tiab(EMDR) OR tiab("Narrative exposure therapy") OR MAINSUBJECT.EXACT("Narrative Therapy") OR tiab(NET) AND pd(20040101-20241231)) OR (tiab(KIDNET) OR tiab("prolonged exposure") OR MAINSUBJECT.EXACT("Prolonged Exposure Therapy") OR tiab("Psychosocial Intervention") OR MAINSUBJECT.EXACT("Psychosocial Interventions") OR tiab("Psychodynamic intervention") OR MAINSUBJECT.EXACT("Psychodynamic Psychotherapy") OR tiab("Mind-Body Therap*") OR MAINSUBJECT.EXACT("Mind Body Therapy") OR tiab("trauma releasing exercise*") |
| Uncertain residence status | Cinahl  Refugee* OR (MH "Refugees") OR asylum* OR (MH "Undocumented Immigrants") OR (MH "Immigrants") OR “uncertain residence status” OR “unaccompanied”  Cochrane Library  Refugee* OR asylum* OR “uncertain residence status” OR unaccompanied OR Immigration OR undocumented migration OR asylum seeking OR political asylum* OR Political refugee* OR Asylum seeker  PubMed  Refugee* OR asylum* OR “uncertain residence status” OR unaccompanied OR Immigration OR undocumented migration OR asylum seeking OR political asylum* OR Political refugee* OR Asylum seeker  PsychInfo  tiab(refugee*) OR MAINSUBJECT.EXACT.EXPLODE("Refugees") OR (MAINSUBJECT.EXACT("Immigration") OR MAINSUBJECT.EXACT("Undocumented Immigration")) OR tiab(asylum*) OR (MAINSUBJECT.EXACT("Political Asylum") OR MAINSUBJECT.EXACT("Asylum Seeking")) OR tiab("uncertain residence status") OR tiab(unaccompanied) |
| PTSD | Cinahl  “PTSD” OR “posttraumatic stress” OR “post-traumatic stress”  posttraumatic stress disorder OR explode(MH "Stress Disorders, Post-Traumatic)  Cochrane Library  PTSD OR “posttraumatic stress” OR “post-traumatic stress”  PubMed  PTSD OR “posttraumatic stress” OR “post-traumatic stress”  PsychInfo  tiab(PTSD) OR tiab("posttraumatic stress") OR tiab("post-traumatic stress") OR MAINSUBJECT.EXACT.EXPLODE("Posttraumatic Stress Disorder") |
